# Supplementary material for: Hybrid Models and Biological Model Reduction with PyDSTool
Source: PLoS Comput Biol. 2012 Aug 9;8(8):e1002628. doi: 10.1371/journal.pcbi.1002628 (PMC3415397; doi:10.1371/journal.pcbi.1002628)
Supplement: Text S4 — Complete source code for the PyDSTool package (version 0.88.120504). Includes API documentation and help files linking to web pages. This file is identical to the current public release on Sourceforge.net. (ZIP) [file pcbi.1002628.s004.zip › PyDSTool/html/PyDSTool.Toolbox.prep_boxplot-module.html]

xml version="1.0" encoding="ascii"?


PyDSTool.Toolbox.prep\_boxplot


| Home | Trees | Indices | Help | PyDSTool |
| --- | --- | --- | --- | --- |

Package PyDSTool ::
Package Toolbox ::
Module prep\_boxplot

[hide private]

[frames] | no frames]

# Module prep\_boxplot

source code


|  |  |
| --- | --- |
| Functions | [hide private] |

|  |  |
| --- | --- |
| prep\_boxplots(data, xlabel\_str, figname='', fignum=1, do\_legend=1, means=1, xlegoff=0, ylegstep=1, ylegoff=0, spacing=None) | source code |

|  |  |
| --- | --- |
| plot\_markers(data, x\_base, name, map, xoff\_sgn, width, off\_fac, markersize) | source code |


|  |  |
| --- | --- |
| Variables | [hide private] |

symbol\_map = `{'isomap': {'K': {10:('ws', 'E'), 50:('ws', 'F'),...`


|  |  |
| --- | --- |
| Variables Details | [hide private] |

### symbol\_map

Value:
:   ```
    {'isomap': {'K': {10:('ws', 'E'), 50:('ws', 'F'), 100:('ws', 'G'), 500
    :('ws', 'H')}, 'eps': {20:('ws', 'I'), 50:('ws', 'J'), 100:('ws', 'K')
    }}, 'pca': {'knee': {1:('wo', 'A'), 2:('wo', 'B')}, 'var': {80:('wo', 
    'C'), 90:('wo', 'D')}}}
    ```

  


| Home | Trees | Indices | Help | PyDSTool |
| --- | --- | --- | --- | --- |

|  |  |
| --- | --- |
| Generated by Epydoc 3.0.1 on Fri May 4 15:24:05 2012 | http://epydoc.sourceforge.net |
